# About the practice of psychiatric euthanasia: a commentary

**DOI:** 10.1186/s12916-017-0896-3

**Published:** 2017-06-27

**Authors:** Jorge Lopez-Castroman

**Affiliations:** 1 Department of Psychiatry, Nîmes University Hospital, 585 Chemin du Mas de Lauze, 30900 Nîmes, France; 2 INSERM u1061, 39 Avenue Charles Flahault, 34090 Montpellier, France; 3 University of Montpellier, 163 Rue Auguste Broussonnet, 34090 Montpellier, France

**Keywords:** Physician-assisted suicide, Palliative care, Psychiatry, Assisted death, Terminal care

## Abstract

Euthanasia motivated by mental disorders is legal in only a few countries and has a short history. In a recent report of all psychiatric euthanasia cases in Belgium between 2002 and 2013, Dierickx and colleagues suggest that the number of these cases is increasing, and provide a profile of the applicants. To date, knowledge of the practice of psychiatric euthanasia is limited, but rising public awareness might increase the number of requests. The authors reveal several shortcomings in cases of psychiatric euthanasia and open avenues for future research.

Please see related article: https://bmcpsychiatry.biomedcentral.com/articles/10.1186/s12888-017-1369-0

## Background

The medical definition of euthanasia is “the act or practice of causing or permitting the death of hopelessly sick or injured individuals in a relatively painless way for reasons of mercy” [1]. Physician-assisted suicide is a modality of euthanasia that involves giving medical assistance to a person requesting to end his or her own life by the self-administration of a lethal mean. Assisted dying, a closely related term, is generally restricted to the prescription of life-ending drugs for terminally ill patients.

The legal regulation of these practices is recent, and definitions remain controversial [2] – probably because debates have focused on the majority of cases involving physical illnesses with short life expectancies. However, in some countries euthanasia is not restricted to the terminally ill, and mental suffering caused by mental disorders can be alleged to request assistance in dying. Of course, strict regulations are followed to ensure that these assisted suicide requests are made voluntarily in the face of untreatable and unrelenting conditions.

There are five other considerations for euthanasia requests: 1) the medical condition conveys unbearable pain; 2) there is no prospect of improvement; 3) available treatments are futile; 4) the person is mentally competent to make a conscious and reasonable choice; and 5) the person is fully informed about the prognosis. For psychiatric cases, assessing fulfillment of these five requirements is problematic.

Not surprisingly, although the medical community largely accepts euthanasia in terminal illness, debate continues on the adequacy of assisted suicide as applied to mental disorders, particularly in treatment-resistant depression [3]. To date, this debate is mostly based on moral or ethical grounds. Objective information about how psychiatric euthanasia takes place might be illuminating. A 2016 report on publicly available cases in the Netherlands [4] revealed that applicants completing a psychiatric assisted suicide were generally affected by chronic mental conditions, often with comorbidities. Prior hospitalizations in psychiatry wards, suicide attempts (often multiple), and personality issues were common, as well as a personal history of traumatic events. For those who work with suicidal patients, or who study suicidal behavior, these features are well known [5].

## New data

The recently published paper by Dierickx et al. [6] describes 179 psychiatric and dementia patients, with no comorbid physical illnesses motivating their request, who were accepted for euthanasia in Belgium between 2002 and 2013. In general, the profile of psychiatric patients in this cohort was similar to those in the study by Kim et al. [4], who found that most psychiatric patients were depressed, middle-aged women, with or without comorbidity. Additionally, approximately one-third of the patients in Dierickx et al.’s cohort were elderly dementia cases.

Some interesting points can be raised from Dierickx et al.’s report. First, consultations by palliative care specialists were not uncommon, and not limited to dementia cases. This is timely, since a distinct field of palliative psychiatry has recently been outlined as a reasonable approach for treating severe persistent mental illness [7]. Second, approximately 1 in 4 patients said that they suffered physical pain, together with psychic pain, despite the absence of reported physical illnesses. It is noteworthy that psychic pain is associated with the modulation of physical pain, and may facilitate suicidal behaviors through increased pain tolerance [8]. Third, although the numbers remain low, Belgium has recently experienced an increase in psychiatric euthanasia cases (0.5% up to 2008, 3% in 2013). A similar trend is observed in the Netherlands up to 2013 [4]. This rise may be associated with increasing public awareness of psychiatric euthanasia.

In cases of mental disorders, physician-assisted death might be justifiable, but only when applicants are fully informed, and have access to adequate treatment options and support in (psychic) suffering [9]. Indeed, psychiatrists should be involved in evaluating euthanasia requests motivated by a mental disorder. This is the case in Belgium, but not in other countries such as the Netherlands or Switzerland. For a given patient, psychiatric assessment may help to ensure that available means are indeed futile to reduce their mental pain and suicidal ideation (which are core symptoms in the suicidal process), and that the person requesting euthanasia is competent and fully informed [10].

The report by Dierickx et al. raises a sensible question about the need for specific criteria and guidelines in assisted suicide for mental conditions. It also reveals some unexplained shortcomings in the practice of psychiatric euthanasia, such as the association of a foreseeable death with mental disorders, or the lack of specialized assessment in some cases. More generally, absence of standardization in the evaluation of psychopathology and mental capacities is problematic. In the Netherlands, a study on psychiatric euthanasia showed that the assessment of decision-making capacity is flawed by the lack of a systematic procedure and disagreements between physicians, which are not uncommon [11].

## Conclusions

Beyond its practical implementation, the debate on euthanasia motivated by mental disorders must be informed by more accurate and detailed records, including standardized methods for diagnosis and capacity assessment, and specific research protocols. For instance, we need to understand how applicants with mental disorders progress through the euthanasia process (e.g., are their therapeutic options reviewed?), and for those whose euthanasia request is denied, how we might attenuate their suffering [12].
